# Ultrasensitive multiplex optical quantification of bacteria in large samples of biofluids

**DOI:** 10.1038/srep29014

**Published:** 2016-07-01

**Authors:** Nicolas Pazos-Perez, Elena Pazos, Carme Catala, Bernat Mir-Simon, Sara Gómez-de Pedro, Juan Sagales, Carlos Villanueva, Jordi Vila, Alex Soriano, F. Javier García de Abajo, Ramon A. Alvarez-Puebla

**Affiliations:** 1 Universitat Rovira i Virgili and Centro de Tecnología Química de Catalunya, Carrer de Marcel·lí Domingo s/n, 43007 Tarragona, Spain; 2 Medcom Advance S.A., Av. Roma, 08840 Barcelona, Spain; 3 Department of Surgery, UD-Vall d’Hebron School of Medicine, Universitat Autònoma de Barcelona, 08035 Barcelona, Spain; 4 Department of Surgery, Hospital el Pilar, 08006 Barcelona, Spain; 5 Department of Clinical Microbiology, Hospital Clinic and School of Medicine, University of Barcelona, Barcelona, Spain; 6 Barcelona Center for International Health Research (CRESIB), Hospital Clínic, University of Barcelona, Barcelona, Spain; 7 Department of Infectious Diseases, Hospital Clínic and School of Medicine, University of Barcelona, Barcelona, Spain; 8 Institut d’Investigacions Biomèdiques August Pi i Sunyer (IDIBAPS), University of Barcelona, Barcelona, Spain; 9 ICFO-Institut de Ciencies Fotoniques, The Barcelona Institute of Science and Technology, 08860 Castelldefels (Barcelona), Spain; 10 ICREA, Passeig Lluís Companys 23, 08010 Barcelona, Spain

## Abstract

Efficient treatments in bacterial infections require the fast and accurate recognition of pathogens, with concentrations as low as one per milliliter in the case of septicemia. Detecting and quantifying bacteria in such low concentrations is challenging and typically demands cultures of large samples of blood (~1 milliliter) extending over 24–72 hours. This delay seriously compromises the health of patients. Here we demonstrate a fast microorganism optical detection system for the exhaustive identification and quantification of pathogens in volumes of biofluids with clinical relevance (~1 milliliter) in minutes. We drive each type of bacteria to accumulate antibody functionalized SERS-labelled silver nanoparticles. Particle aggregation on the bacteria membranes renders dense arrays of inter-particle gaps in which the Raman signal is exponentially amplified by several orders of magnitude relative to the dispersed particles. This enables a multiplex identification of the microorganisms through the molecule-specific spectral fingerprints.

Septicemia affects nearly 20 million people per year with a mortality rate of 30–40%^1^,^2^. These patients require intensive care with associated high costs, which impose significant health-care, economic, and social burdens. In particular, each septic patient in the United States incurs costs of approximately $25,000 during hospitalization, totalling a nationwide annual bill in excess of $17bn^2^. The time to initiation of effective antimicrobial therapy is known to be the single strongest predictor of outcome, as every hour delay in its administration increases by 8% the risk of death^3^,^4^. Besides the obvious economic benefits, the development of fast, accurate, and inexpensive diagnostic methods thus appears as a major goal for alleviating human pain. Microbial culture remains the most widespread technique for identifying the infectious agent, but unfortunately it requires 24–72 hours to provide a conclusive diagnosis for common infections^5^. Understandably, a large deal of work has been devoted over the last three decades to developing alternative methods for fast identification of bacteria in suspected patients^6^. These methods include immunology-based approaches (e.g., enzyme-linked immunosorbent assay (ELISA), as well as fluorescence and radio immunoassays)^7^, nucleic acid identification (e.g., polymerase chain reaction, PCR)^8^, and, lately, spectrometry-based procedures (e.g., matrix-assisted laser desorption/ionization-time-of-flight, MALDI-TOF, mass spectrometry)^9^. Although generally faster than just sequential microbial culture, these techniques still require hours to days, depending on the pathogen. Additionally, immunological and nucleic-acid tests are expensive (around $200 each) and monoplex (one test per target microorganism), while MALDI-TOF still relies on microbial culture to isolate pure colonies. As a consequence, a cocktail of broad-spectrum antibiotics is generally recommended to cover all potential pathogens until obtaining a conclusive identification. Apart from its inherent cost and adverse health effects, this indiscriminate use of antibiotics induces bacterial resistance^10^, a growing problem of modern pharmacopeia^11^. Despite recent advances in fast recognition of bacterial resistance, society urges for the development of new diagnostic systems capable of providing fast, accurate, inexpensive, and if possible multiplexed identification of infectious agents in body fluids that will yield a rapid and guided treatment that avoids the use of spurious drugs^12^,^13^.

Recent advances in nanoscience, spectroscopy, magnetism, plasmonics, and microfluidics^8^,^14^,^15^,^16^,^17^,^18^ have generated great expectations for the development of new approaches to bacteria characterization^19^ and detection^20^,^21^,^22^,^23^,^24^,^25^, especially with the use of nano and microparticles^26^. Unfortunately, the methods so far proposed are generally time consuming, capable of only exploring small sample volumes (~microliters) not relevant for clinical diagnosis of septicemia^4^,^18^, working exclusively for one *a priori* selected pathogen^20^,^27^, not truly multiplex^21^, requiring from multiple external labels^22^, or relying on additional steps to record a suitable signal for identification^18^,^23^. Here, we report a microorganism optical detection system (MODS) and demonstrate exhaustive pathogen identification through fast screening of large body-fluid volumes (milliliters of blood) for bacterial content, down to the single colony forming unit detection, as required by standard medical practice for the analysis of biological samples^5^. Specifically, we achieve detection and quantification of bacteria in real time and in a multiplexed manner.

## Results

### Microorganism optical detection system (MODS)

**Figure 1:**
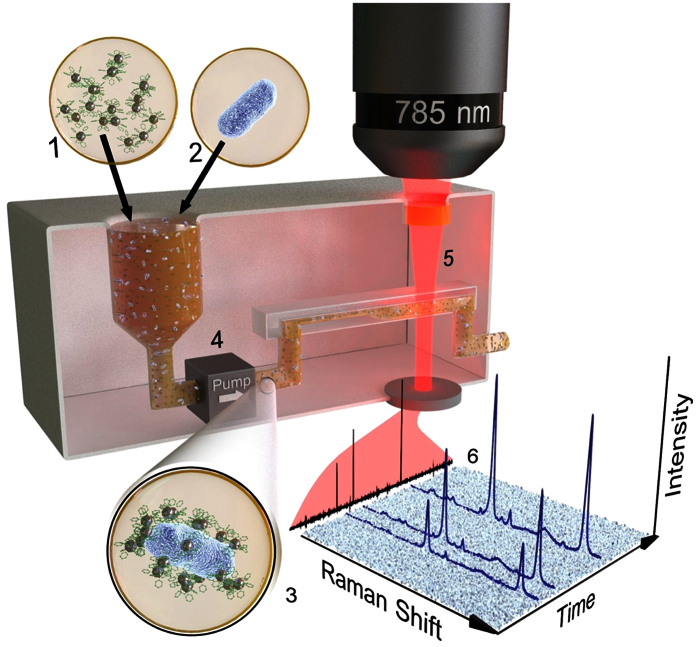
Microorganism optical detection system (MODS). Conceptual view of MODS and its relevant components. Silver nanoparticles (NPs) are separately labelled with different Raman-active molecules and functionalized with bacteria-selective antibodies (**1**). A nanoparticle dispersion is mixed in a vessel (3 mL) with the sample fluid, possibly infected (**2**). Several types of bacteria are targeted using NPs prepared with different specific combinations of Raman molecules and antibodies. The presence of one of these microorganisms induces aggregation of antibody-matching NPs on its membrane, rapidly evolving towards full random coverage (**3**). The mixture is circulated through a millifluidic channel with a micropump (**4**) and passing through the focus of a 785 nm laser (**5**), which is in turn spectrally analysed to record the SERS signal generated by the Raman-active molecules (**6**). Targeted bacteria produce a large increase in SERS signal, whose spectral fingerprints allow us to identify the type of pathogen.

**Figure 2:**
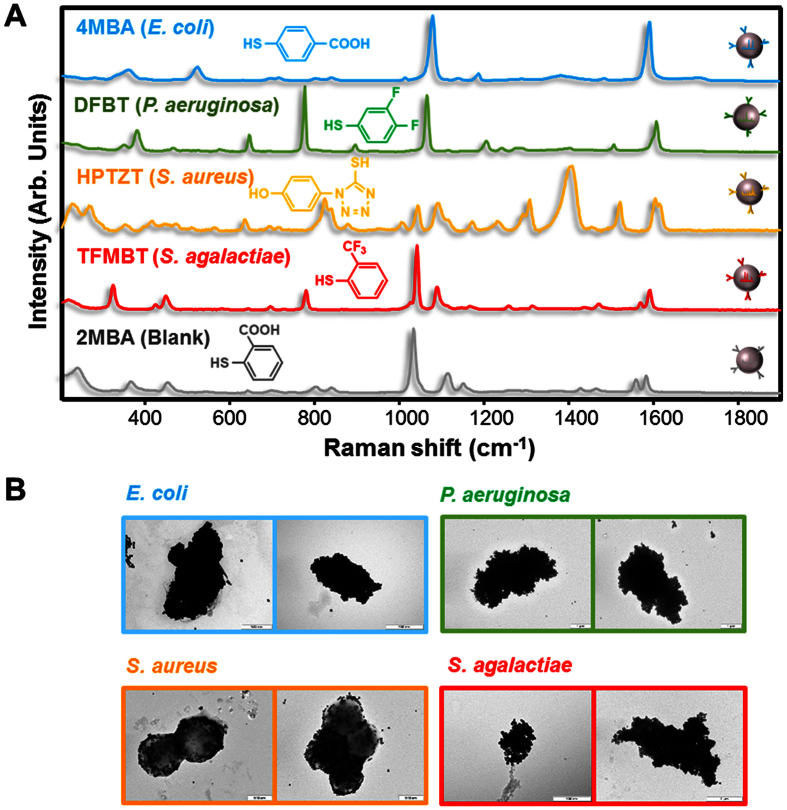
Encoded nanoparticles and their interaction with bacteria. (**A**) SERS spectra of the different coded particles here used for each targeted pathogen, along with their corresponding labelling molecules. (**B**) Transmission electron microscope images of the targeted bacteria (*E. coli*, *P. aeruginosa, S. aureus*, and *S. agalactiae*) coated with their respective matching NPs.

Motivated by the need for an accessible, highly sensitive, and selective platform for the screening of pathogens in large samples of biological fluids (i.e., serum or blood), we have engineered a device as described in Fig. 1. Our MODS device relies on the use of plasmonic nanoparticles (NPs) coded with Raman-active molecules and functionalized with selective antibodies. In contrast to previous demonstrations of *in vivo* imaging using silica or polymer protected nanoparticles^28^, our plasmonic colloids are protected with a thin layer of 11-mercaptoundecanoic acid (MUA, ~1 nm) in order to facilitate their plasmonic interaction in between particles. For each targeted pathogen receptor, we prepare NPs (silver spheres, ~60 nm diameter) with a unique combination of Raman label and selective antibody. The NPs produce a relatively weak Raman signal when they are dispersed in a fluid. In contrast, the presence of one of the targeted pathogens triggers the accumulation of its partner NPs on the antigen-carrying membrane of the microorganism, rapidly reaching full random coverage^29^. Multiple gaps between NPs are then formed, which act as optical hotspots in which Raman scattering is enhanced by several orders of magnitude relative to the same number of noninteracting NPs^30^,^31^,^32^,^33^. The resulting surface-enhanced Raman scattering (SERS)^34^ signal is sufficiently intense as to allow us to record pathogen-specific inelastic light spectra (Fig. 2A) from the NP-covered bacteria (Fig. 2B). By driving the sample through a millifluidic channel (0.4 mm ×2 mm section, aimed by a micropump, see Fig. 1A and Supplementary Figure S1), where a backscattered detecting laser (785 nm) continuously monitors the liquid stream (one spectrum every 270 ms over an illuminated volume of ~0.32 μL), we successfully and simultaneously quantify multiple different types of bacteria at a pace of 13 minutes per mL of blood or serum. Importantly, this method can be readily scaled to cope with many more pathogens including viruses or eukaryotic cells such as fungi or protozoa, in a single pass without increasing the sampling time, simply by preparing NPs functionalized with more combinations of Raman labels and selective antibodies or aptamers.

### MODS demonstration in serum

For demonstration of bacteria detection, we prepared five dispersions of coded NPs, each of them functionalized with a different aromatic thiol that yields a unique Raman spectrum (Fig. 2A and Supplementary Figure S2). The coded NPs were subsequently and separately functionalized with the corresponding membrane-selective antibody, at low concentration, for recognition of *E. coli* and *P. aeruginosa* (gram negative rod-like bacteria), as well as *S. aureus* and *S. agalactiae* (gram positive spheroidal cocci). The fifth NP dispersion was functionalized with serum albumin and used as a blank. All five NP dispersions were then mixed in the MODS vessel (Fig. 1) at a concentration of ~10^7^ NPs per mL per Raman code, which was found to be optimum for yielding fast NP-pathogen attachment with a minimum background SERS signal of unattached NPs (see Supplementary Figure S3).

**Figure 3:**
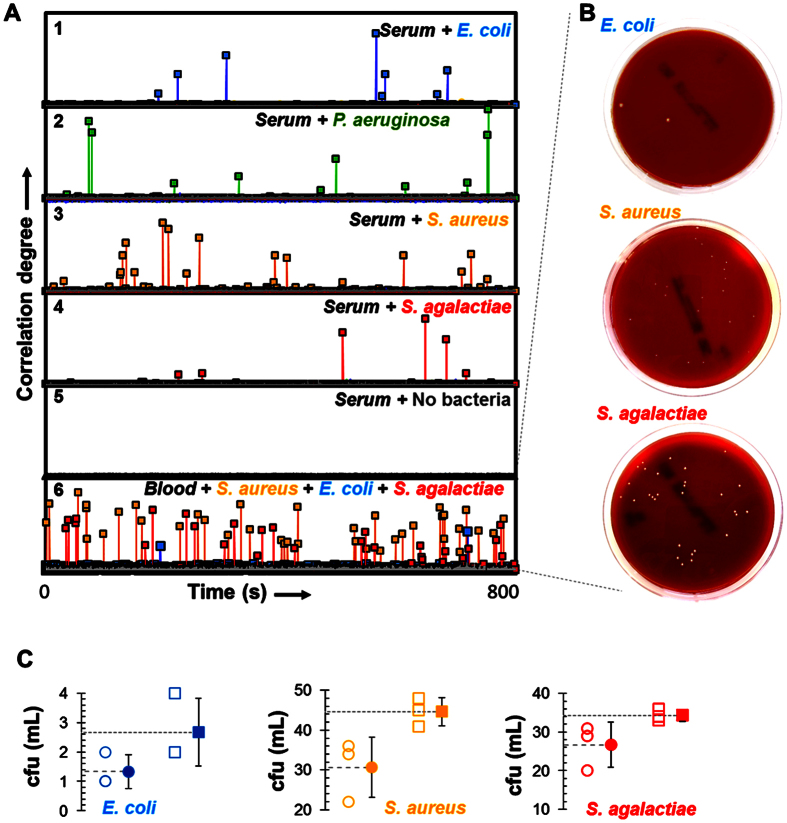
MODS performance for contaminated samples. (**A**) Correlation between a temporal series of spectra collected over 270 ms intervals and the SERS reference of the labelled NPs (Fig. 1B). The analysed serum samples contain either one pathogen (1–4, see labels) or no pathogen (5, blank). Series 6 shows the result for a blood sample spiked with a combination of three different bacteria and concentrations (*S. aureus*, *E. coli*, and *S. agalactiae*). Large correlation values reveal the passage of an individual bacteria or CFU. (**B**) Bacterial cultures (24–48 hours) for the microorganism inoculated in the blood samples (series 6). White spots correspond to CFUs. (**C**) Comparison of the bacteria concentrations (CFUs per mL) as determined by MODS (open squares) for the sample contaminated with three pathogens (series 6) versus traditional cultures (open circles). Averages over three runs of both MODS and culture experiments are shown by the corresponding solid symbols.

Further, to limit the formation of spurious aggregates in the colloidal solution, which may lead to false positives, we added to the mixture twice as many non-coded BSA-protected NPs to the mixture. As a first demonstration, serum contaminated with only one of the four mentioned bacteria was then added to the MODS mixing vessel, and a time series of SERS spectra were collected as the mixture was circulated through the laser focus (Fig. 3A and Supplementary Figures S4–S7). The majority of the time steps only sampled dispersed NPs, which produced a weak or none SERS signal. Occasionally, a targeted colony forming unit (CFU) traversed the laser focus, giving rise to ~10^3^ higher SERS signal due to the concentration of particles on the bacterial surface and the subsequent formation of NP gaps, as discussed above. We then calculated the correlation of the recorded spectra with the reference ones for each of the Raman codes (Fig. 2A), and obtained clear identification of the added pathogen. The experiment was successfully repeated for samples containing mixtures of two bacteria (Supplementary Figure S8). Importantly, we note that no false identifications were observed in any of the analyses (i.e., no signatures from the bacteria types that were absent from the serum), and no positives were recorded from blank samples (Fig. 3A and Supplementary Figures S4–S9). Incidentally, a significant dispersion in correlation was observed (i.e., absolute SERS intensity per recognition event), which we attribute to the size of the detected CFUs, ranging from single cells to larger colonies (Supplementary Figure S10). Remarkably, all of the obtained concentrations were consistent with those found using conventional bacterial culture, but with a considerably lower standard deviation (Supplementary Figures S4–S9).

### MODS demonstration in blood

We demonstrated selectivity and multiplexing of MODS in whole blood samples by contaminating blood simultaneously with *S. aureus*, *E. coli*, and *S. agalactiae* in different concentrations ranging from units to tens of CFUs per mL. It is important to note that blood samples spiked with microorganisms constitute a good model to emulate bacterial infection in actual patients, as an infection in the latter originates in spiking of biological fluids from an external source. MODS analysis of this sample revealed all three pathogens (Fig. 3A and Supplementary Figure S9), while a statistical analysis of the results based on three runs of the experiment (Fig. 3C) determined their respective concentrations in excellent agreement with bacterial cultures (Fig. 3B and Supplementary Figure S9). Incidentally, positive events varied in the degree of correlation, as expected from the bacterial diversity (Supplementary Figure S10). We stress that each MODS analysis took 13 min, which is a substantial reduction in time compared with bacterial cultures (24–72 hours). These results further demonstrate the ability of MODS to accurately resolve pathogen concentrations in complex samples infected simultaneously with different pathogens at very different concentrations in a single pass, as illustrated in Fig. 3 and Supplementary Figure S9, where *E. coli* is present in a much lower density than *S. aureus* and *S. agalactiae*.

### Nanoparticle aggregation on bacteria

**Figure 4:**
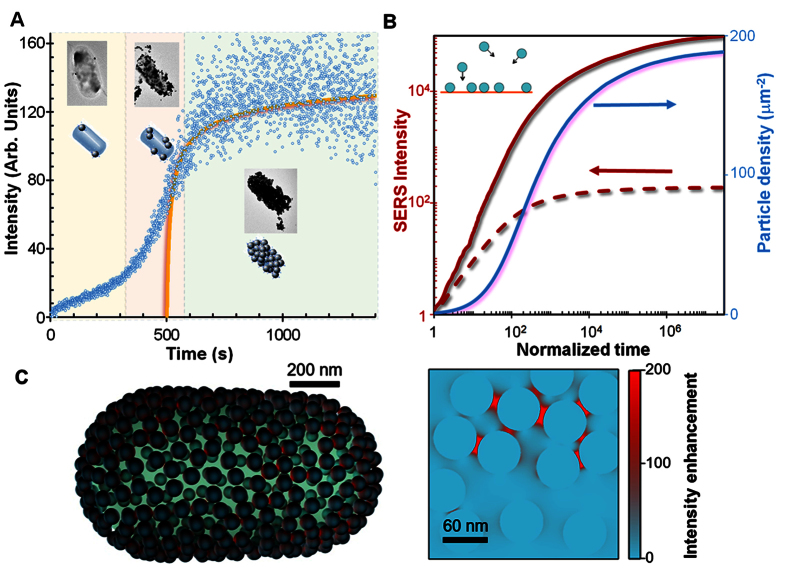
Kinetics of sensing enhancement through NP aggregation. (**A**) Kinetics of NP aggregation as measured through the time-dependent SERS signal (symbols) after adding *E. coli* to the mixture of coded NPs. Solid curve: theory from (**B**). (**B**) Simulation of the temporal evolution of 60 nm Ag NP aggregation on the bacteria membrane produced by random NP-membrane encounters, resulting in a rapidly growing NP density (right scale) and SERS intensity (left scale, calculated per μm^2^ of membrane area and normalized to the signal from an individual NP). The latter is given with (solid curve) and without (broken curve) inclusion of the effect of NP gap hotspots. The time is normalized to the average delay interval between consecutive NP arrivals. The theory curve in A is scaled to 17.3 arrivals per second, as estimated from kinetic theory (see ESI). (**C**) Near-electric-field intensity in a rod-like individual *E. coli* covered with Ag NPs (top, intensity plotted on the NP surfaces) and detail of the array (bottom, intensity on a surface passing by the NP centers), revealing the formation of optical hotspots. The intensity is averaged over light incidence directions and polarizations, the light wavelength is 785 nm, and the colour scale is saturated to improve visibility.

The kinetics of functionalized NP aggregation on the pathogen membrane is a key factor of MODS. As a representative example, after adding a large concentration of *E. coli* to the pool of five coded NPs (time 0), we found three distinct stages in the temporal evolution of the resulting SERS signal (Fig. 4A). First, NP aggregation is initially very slow due to the relatively infrequent NP-bacteria encounters before sample and NPs fluids are fully intermixed, yielding just a slow increase in Raman signal; second, diffusion eventually brings the NPs closer to the bacteria after ca. 300 s, resulting in more frequent encounters and a faster linear increase in SERS intensity; finally, the signal eventually reaches a plateau after ca. 700 s, consistent with previous literature^35^, indicating saturated coverage of the membrane. The latter stage is also affected by signal depletion produced by flocculation, as the increased weight of NP-covered CFUs pulls them away from the laser focus. Insight into these experimental results is provided by a Monte Carlo simulation of particle sticking (Fig. 4B, right scale), which reveals a characteristic saturation at a NP random coverage ~60% of the maximum close-packed density^29^. A large occurrence of NP gaps takes place at high NP coverage (Supplementary Figure S13). Although the laser wavelength is far from the NP plasmons (Supplementary Figures S2 and S11), these gaps produce a dramatic enhancement in the Raman signal (Supplementary Figure S12). Averaging over the gap distribution (Supplementary Figure S14), we find an increase in SERS intensity by 3 orders of magnitude compared with non-interacting NPs (cf. solid and broken red curves in Fig. 4B, left scale). The signal resulting from positive CFU encounters is further amplified by the effect of attached NP accumulation. We note that the modelled temporal evolution of the SERS signal agrees rather well with the measured kinetics (Fig. 4A), without adjustable parameters other than the time after which sample and NPs fluids are considered to be fully intermixed (~500 s).

### Optical effects of the nanoparticle aggregation on bacteria

Particle accumulation, gap formation, and the resulting SERS enhancement are thus pivotal elements that allow us to obtain intense signals from bacteria content well above the background of dispersed NPs. In support of this conclusion, we further simulate the near-electric-field light intensity at 785 nm wavelength for a rod-like structure mimicking an *E. coli* specimen (Fig. 4C), which reveals large enhancements at the gaps between neighbouring NPs, accompanied by relatively weak modulations away from the gaps (see also Supplementary Figure S14). Additional simulations of SERS enhancement for different particle compositions and sizes (Supplementary Figure S15) suggest that an improvement in sensitivity can be gained by optimizing these parameters. In particular, the optimum particle size may depend on the dimensions and morphology of the pathogen and its affinity for the NP ligand biomolecules, as the figure of merit for MODS is the ratio of the SERS signal coming from the microorganisms to that originating in the dispersed NPs.

## Discussion

We have developed an infection diagnostic method and device (MODS) that can safely identify and quantify, with sensitivity down to the single CFU, the presence of bacterial pathogens in biological fluids, specifically in serum and blood. The method can screen milliliters of liquid at a rate close to 10 min/mL. Comparison of MODS results with those obtained from bacterial culture shows that although bacterial amount is usually slightly larger for MODS, its standard deviation is consistently smaller. Further, all the results, for both techniques, show the same order of magnitude of bacteria, which is the relevant parameter in clinical diagnosis^36^,^37^. Although here we demonstrate the concept with four bacterial agents, the detection method can be expanded to simultaneously identify many more of them by using the large number of available spectral codes and selective antibodies/aptamers. Indeed, we have previously demonstrated a method for the codification of nanoparticles with almost any molecule that shows intense SERS signal^38^. Additionally, antibodies used in common clinical practice, such as those developed for immunology, can be readily incorporated in MODS. However, if required for unknown/emerging diseases, methods such as the systematic evolution of ligands by exponential enrichment (SELEX)^39^ may render new reliable selective antibodies/aptamers. Furthermore, although MODS has been demonstrated here only with bacteria, the method has the potential to also analyse other microorganisms such as viruses, protozoa, fungus, and neoplastic cells in body fluids within minutes.

Early diagnostic of sepsis in blood is a pressing clinical need. Even though MODS is not yet capable of discerning in between resistant and non-resistant microorganisms directly, it offers an accurate diagnostic in very short time as compared with all other currently available methods. Further, MODS cannot only guide in the choice of an accurate treatment for a given patient, but it can also monitor the effect of that treatment on the time evolution of the infection. Additionally, the method can be used to test the efficiency of antibiotics by just adding different drugs to different aliquots of the patient sample with the subsequent determination of the bacterial population in the samples with MODS. These characteristics render our technology of great interest for both the accurate diagnostic of patients and the screening of large populations, especially in suspected pandemics.

With a growing population of patients at risk of developing infections, a highly sensitive, nonsurgical, nonradioactive method for repeated monitoring should be clinically useful. Thus, joining a list of next-generation diagnostics (MALDI-TOF, lateral-flow, PCR), MODS aims to build toward early identification of infectious disease that may result in improved patient outcomes and decreased treatment toxicities. Further, MODS may be amenable to further engineering to also allow monitoring of ubiquitous fluids such as air and natural waters for assessing the microbiological quality of human environments.

## Methods

### Materials

Silver nitrate (99.99%, AgNO_3_), trisodium citrate dihydrate (≥99.5%), L-ascorbic acid (≥99.0%), magnesium sulfate (≥99.0%, MgSO_4_), ethanol (99.5%, EtOH), 11-mercaptoundecanoic acid (95%, MUA), 4-mercaptobenzoic acid (99%, 4MBA) 2-mercaptobenzoic acid (97%, 2MBA), 3,4-difluorobenzenethiol (96%, DFBT), 2-(trifluoromethyl)benzenethiol (96%, TFMBT), 1-(4-hydroxyphenyl)-1*H*-tetrazole-5-thiol (97%, HPTZT), bovine serum albumin (≥98.0% , BSA), *N*-(3-dimethylaminopropyl)-*N′*-ethylcarbodiimide hydrochloride (BioXtra, EDC), sodium chloride (BioXtra, ≥99.5%, NaCl), Dulbecco’s phosphate buffered saline (D8537, DPBS), and human blood (BCR634 FLUKA) were purchased from Sigma-Aldrich. All reactants were used without further purification. MilliQ water (18 MΩ cm^−1^) was used in all aqueous solutions, and all the glassware and magnetic stirrers were cleaned with aqua regia and with a potassium hydroxide solution in isopropanol/water before all the experiments.

### Antibodies and Bacteria

Antibodies selected for this study are the same that those used in the clinical practice for the immunological methods. *Escherichia coli* (ab30522, *E. coli*) and *Streptococcus agalactiae* (ab41203, *S. agalactiae*) antibodies were purchased from Abcam. *Pseudomonas aeruginosa* (MA1-83430, *P. aeruginosa*) and *Staphylococcus aureus* (MA1-83467, *S. aureus*) antibodies were purchased from Life Technologies. Enriched thioglycollate medium (221742, BBL) was purchased from BD (Becton, Dickinson and Company) and Columbia agar + 5% sheep blood plates (43 041) were purchased from bioMérieux. Bacterial samples were obtained from the Department of Clinical Microbiology, Hospital Clinic, Barcelona, Spain.

### Synthesis of citrate-stabilized spherical silver nanoparticles

Spherical silver nanoparticles (Ag NPs) of approximately 62 nm in diameter were produced by a combination of previously reported approaches^38^,^40^,^41^,^42^. Briefly, MilliQ water (250 mL) was heated under vigorous stirring. A condenser was used to prevent solvent evaporation. Next, aqueous solutions of trisodium citrate (3.41 mL, 0.1 M) and ascorbic acid (0.25 mL, 0.1 M) were consecutively added into the boiling water. After 1 min, a premixed aqueous solution containing AgNO_3_ (0.744 mL, 0.1 M) and MgSO_4_ (0.56 mL 0.1 M), which was previously incubated at room temperature for 5 min, was rapidly injected into the reaction vessel under vigorous stirring. The colour of the solution quickly changed from colourless to yellow and then gradually into dark orange. Boiling was continued for 1 h under stirring to ensure the completeness of the reaction. The silver concentration of the synthesized particles was 2 × 10^−4 ^M (i.e., ~10^10 ^NPs per mL).

### Mercaptoundecanoic (MUA) acid functionalization and codification of Ag NPs

After NP synthesis, MUA was used in order both to provide colloidal stability to the Ag NPs during the encoding process and later on to use the carboxylic functionality for the coupling of the antibodies. Specifically, five aliquots (25 mL each) of the synthesized Ag NPs were cleaned by centrifugation (5400 rpm, 30 min) and redispersed via sonication (during 5 min) in a solution containing MilliQ water (3.27 mL) and EtOH (21.67 mL). Subsequently, the Ag NPs were functionalized with a small amount of MUA (2.4 molecules/nm^2^) by rapidly adding a solution containing MUA (24.76 μL, 1.0 × 10^−3 ^M in EtOH) and NH_4_OH (30 μL, 29% aqueous solution) to each aliquot under vigorous stirring. Agitation was continued for 28 h to assure the complete MUA functionalization on the silver surface. Finally, the MUA functionalized Ag NPs aliquots were encoded with five different Raman labels (1.6 molecules per nm^2^ of 4MBA, 2MBA, DFBT, TFMBT, and HPTZT, respectively). To this end, the stock solutions (16.51 μL of a 10^−3 ^M) of the five different SERS codes were added to the aliquots under strong magnetic stirring. Once again, stirring was continued for another 28 h. The encoded Ag NPs solutions were then centrifuged and redispersed twice in MilliQ water to remove the excess of NH_4_OH, EtOH, and any unreacted Raman label prior to antibody coupling. The concentration of NPs was calculated for each aliquot using the Lambert-Beer law and an extinction coefficient of 7.79 × 10^10 ^M^−1 ^cm^−1^, derived from literature^43^ and adjusted to 0.14 nM for all of them.

Additionally, another aliquot (25 mL) of the synthesized Ag NPs was cleaned by centrifugation (5400 rpm, 30 min) and redispersed in MilliQ water (25 mL). The Ag NPs were then functionalized with MUA by adding, under vigorous stirring, 24.76 μL (1.0 × 10^−3 ^M in EtOH). Agitation was continued for 28 h to ensure the complete MUA functionalization on the silver surface. Finally, Ag NPs were further modified with BSA (41.26 μL, 1.0 × 10^−3 ^M in MilliQ water) under magnetic stirring for 24 h, followed by one cleaning step centrifugation to remove excess of BSA and any unreacted MUA molecules (5400 rpm, 30 min, redispersed in MilliQ water). The concentration of these NPs was calculated and adjusted to 0.034 nM.

### Antibody conjugation to AgNPs

200 μL of DPBS and EDC (39.2 μL, 250 nM in DPBS) were added over each codified Ag NPs solution (1 mL, 0.14 nM, Raman labels: 4MBA, 2MBA, DFBT, TFMBT, and HPTZT), the mixtures were shaken for 5 min at room temperature, and then the antibodies solutions were added (1.46 μL, ~6.67 μM, *E. coli*, BSA, *P. aeruginosa*, *S. agalactiae*, and *S. aureus*, respectively). The resulting mixtures were first shaken for 2 h at room temperature, then cleaned twice by centrifugation to remove the excess of unreacted EDC and antibodies (3000 rpm, 10 min), and redispersed in DPBS/MilliQ water (1:3). The concentration of each Ab-modified Ag NPs solution was measured and adjusted to 0.14 nM. The resulting NP solutions were stored at 4 °C.

### Characterization of the nanoparticles

UV-Vis spectroscopy (Lambda 19, PerkinElmer) and transmission electron microscopy (TEM, JEOL JEM-1011 operating at 100 kV) were used to characterize the optical response, structure, and size of the nanoparticles during the functionalization process. To characterize the codification process, SERS spectra were collected in backscattering geometry with a Renishaw Invia Reflex system equipped with a 2D-CCD detector. The spectrograph used a high resolution grating (1200 g cm^−1^) with additional band-pass filter optics. A 785 nm diode laser was focused onto the colloidal solution ([Ag^0^] = 0.1 mM) by a long-working distance objective (0.17 NA, working distance 30 mm). The spectra were acquired with an exposure time of 100 ms (depending on Raman intensity saturation) and a laser power at the sample of ca. 300 mW.

### Microfluidic device manufacturing

The microfluidic device was fabricated with polydimethylsiloxane (PDMS) by replica moulding using an aluminum master mold. PDMS (Sylgard 184, Dow Corning) was prepared by mixing pre-polymer and curing agent at a standard 10:1 ratio. The mixture was poured onto the mold, degassed in vacuum, and cured at 80 °C. After one hour, PDMS was peeled off the mold and the fluidic access holes (inlet and outlet) were punched. The final device was obtained by bonding the PDMS layer to a slide cover (130–170 μm-thick) after oxygen plasma treatment (100 W, 3% O_2_, 0.2 mbar, 40 s) (Plasma Flecto 10, Plasma technology GmbH). The microfluidic device consisted of a single channel with variable dimensions. The inlet channel had a width of 200 μm and a depth of 100 μm, which was enlarged to 400 μm-width and 2 mm-depth in the outlet channel (i.e., the optical detection section, see Supplementary Figure S1).

### Bacterial samples

Bacteria were inoculated in enriched thioglycollate medium (BBL), incubated at 37 °C for 18 h, and then diluted with saline solution (NaCl 0.9%). Next, the required amount of bacterial solution, to reach the desired final concentration, was added into the corresponding fluid, that is, saline solution or human blood containing Ab-modified Ag NPs (1 μL of each encoded particle per mL of sample) and twice as many serum-albumin protected unlabelled AgNPs as the total amount of codified AgNPs. Bacterial culture is the gold standard for bacterial quantification in environmental and clinical microbiology. Essentially, it is based in the spread of a known volume of the target liquid onto a blood agar plate. After some time (24–48 h for most bacteria) the single colony forming units (CFUs), dispersed in the liquid, multiply in the blood agar plate to form colonies that can be counted visually. Each colony is equivalent to a single CFU in the initial bacterial solution^36^,^37^. Thus, to verify the final bacterial concentration per sample, several aliquots of each solution were spread in agar-blood plates and incubated for 24–48 h at 37 °C. After this time, the number of colonies in each plate was counted in order to calculate their concentration of CFU per mL. To further corroborate the interaction between the Ab-modified Ag NPs with the corresponding bacteria, equal volumes of Ag NPs (0.14 nM) and bacterial solution (~10^6^ CFU mL^−1^) were mixed, and incubated for 15 min at 37 °C. Small fractions (10 μL) of these mixtures were deposited on carbon coated copper grids and the samples were allowed to dry before performing TEM analysis.

### Measurement system setup

The PDMS microfluidic device was integrated in a stage comprising an inlet vessel with the precharged NPs where the sample is placed, a micropump (mp6, Bartels) and an outlet vessel. All these elements were connected with polytetrafluoroethylene (PTFE) 0.8 mm wide tubes. The flow rate was controlled with a Bartels extend Micropump Controller and set to 1.74 mm s^−1^. SERS measurements were collected with a Renishaw Invia Reflex. The laser (785 nm, 300 mW) was focused with a macro-objective (6 mm aperture and −18 mm focal distance), providing an efficient spot of 0.5 mm. The scan collection time was set to 270 ms per spectrum, providing an acquisition speed of 1.85 mm s^−1^, which results in an evaluation speed of 13.3 min per mL of sample, during which 3000 scans are obtained. Data deconvolution was carried out by principal component analysis and classical least squares using the Wire 4.1 software from Renishaw.

### Electromagnetic simulations

The simulation of the electric near-field and SERS enhancements in Fig. 4C and Supplementary Figures S11, S12 and S15 were carried out with a fully converged multiple elastic scattering of multipolar expansions (MESME) method^44^. In particular, the SERS enhancement at a given position was approximated as the product of the field enhancements calculated at that position for wavelengths corresponding to the incident and emitted light. Each of these field enhancements was averaged over incidence light directions and polarizations before multiplying them to yield the SERS enhancement. The field enhancement of Supplementary Figure S14 was obtained using the boundary-element method (BEM)^45^. The dielectric functions of gold and silver used in these simulations were taken from tabulated measurements^46^. The media inside and outside the bacteria were both assumed to have the permittivity of water, ε = 1.77.

### Simulation of particle attachment

We simulated the process of NP attachment on the membrane of the targeted bacteria by implementing a Monte Carlo method in which particles landed on the microbe surface at random spots, following either randomly oriented trajectories (Fig. 4B, and solid curves in Supplementary Figures S13 and S14 left) or approaching it along the surface normal (dashed curves in Supplementary Figures S13 and S14 left). Particles colliding with any previously stuck particle were disregarded. The probability distribution of randomincidence orientations was taken to be proportional to sin θ cos θ as a function of incidence angle θ relative to the surface normal, where the cosine function reflects the average particle flux for a surface tilted by that angle, whereas the sine function comes from the Jacobian of spherical coordinates. Statistical analysis of the particle positions produced by these simulations allowed us to obtain the time-dependent particle density (Fig. 4B) and the distribution of inter-particle gap distances (Supplementary Figure S13). Each of the results here presented was averaged over 10^5^ simulation runs for a square membrane of large side compared with the particle diameter. Opposite sides of the square were identified (toroidal topology) to minimize edge effects. Convergence was achieved for a side length ~20 particle diameters.

In the resulting Figures S13 and 14 left, the time axis is expressed in units of the average interval between collisions over an area equal to the unit cell of a closed-packed arrangement of the NPs (i.e., a time unit is defined as the average time needed to have one particle colliding for each element of area equal to 3^1/2^*D*^2^/2, where *D* = 60 nm is the particle diameter). For the sake of readability, the time in Fig. 4B is instead normalized to the average interval separating consecutive particle collisions on an area of 1 μm^2^. Finally, for the comparison with experiment in Fig. 4A, the theory curve is scaled to have 17.3 collisions per second per μm^2^, as predicted by the impingement rate equation^47^.


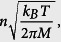


where *n* = 10^9^ NP/mL is the particle density (notice that the NP dispersion used to study the kinetics in Fig. 4A was diluted with respect to the cocktail used for MODS analyses), *T* = 300 K is the temperature, and M = 2.2 × 10^−18 ^kg is the NP mass corresponding to 60 nm silver spheres. Although this equation is a result of frictionless kinetic theory, we apply it here assuming that the collision rate is dictated by microscopic random displacements aimed by Brownian motion.

### Simulation of SERS enhancement produced by particle attachment

We obtained the temporal evolution of the SERS signal (Fig. 4B) by combining the gap-mediated SERS enhancement (Supplementary Figure S12) and the time-dependent particle (Fig. 4B) and gap (Supplementary Figure S13) densities. The SERS enhancement was calculated from the weighted contributions of both the individual NPs (Supplementary Figure S12, dashed lines) and the gaps formed between them (Supplementary Figure S12, solid curves). Incidentally, multiple inter-particle interactions beyond dimers formed by nearest neighbours have a relatively weak effect (Fig. 4C and Supplementary Figure S14), which should be further reduced when averaging over random NP arrangements and light incidence directions and polarizations. Consequently, we approximated the SERS enhancement at the gaps by simulating isolated dimers (Supplementary Figure S12). For simplicity, we considered a zero Raman shift in our simulations (i.e., we used the black solid curve of Supplementary Figure S12). We found the effect of finite Raman shift to be marginal, as the particles were operating at an off-resonance wavelength ≥ 785 nm (see Supplementary Figure S11). Incidentally, the SERS enhancement of Fig. 4B is normalized to the emission from an individual NP.

Simulation of the effective permittivity of the NP coating described as a metamaterial. In order to quickly assess the effect of the NP coating on the near-field at the large scale commensurate with the microbe, we assimilated the NPs to an equivalent homogeneous thin film with an effective dielectric function calculated in such a way that the film had the same normal-incidence reflection coefficient as a layer of randomly distributed particles (i.e., the layer was treated as a metamaterial). We estimated the effective dielectric function from the dipoles induced per gap and per particle, as obtained from electromagnetic simulations of the gap-size-dependent polarizability of NP dimers using MESME. The effective dielectric function was then expressed as ε + (4π*/t*)*β*, where *β* is the sum of NP and gap polarizabilities normalized per film-surface area, *t* is the equivalent film thickness, and ε = 1.77 is the permittivity of the surrounding medium (water). The dependence on the choice of *t* was found to be very mild, and actually, the results obtained for the near-field distribution calculated with *t* = 10 nm and *t* = 20 nm were nearly indistinguishable on the scale of Supplementary Figure S14 right.

## Additional Information

**How to cite this article**: Pazos-Perez, N. *et al*. Ultrasensitive multiplex optical quantification of bacteria in large samples of biofluids. *Sci. Rep*. **6**, 29014; doi: 10.1038/srep29014 (2016).

## Supplementary Material

Supplementary Information
