# Supplementary material for: Ultrasensitive multiplex optical quantification of bacteria in large samples of biofluids
Source: Sci Rep. 2016 Jul 1;6:29014. doi: 10.1038/srep29014 (PMC4929498; doi:10.1038/srep29014)
Supplement: Supplementary Information [file srep29014-s1.pdf]

Supplementary information:

Ultrasensitive multiplex optical quantification of bacteria in large samples of biofluids

Nicolas Pazos-Perez,[1,2] Elena Pazos,[2] Carme Catala,[1,2] Bernat Mir-Simon,[2,3] Sara Gomez-de-Pedro,[2] Juan Sagales,[2] Carlos Villanueva,[2,4] Jordi Vila,[5,6] Alex Soriano,[7,8] F. Javier García de Abajo,[9,10,*] Ramon A. Alvarez-Puebla[1,2,10,*]

[1] Universitat Rovira i Virgili and Centro de Tecnología Química de Catalunya, Carrer de Marcellí Domingo s/n, 43007 Tarragona, Spain.

[2] Medcom Advance S.A., Av. Roma, Barcelona, 08840 Spain.

[3] Department of Surgery, UD-Vall d'Hebron School of Medicine, Universitat Autònoma de Barcelona, 08035 Barcelona, Spain.

[4] Department of Surgery, Hospital el Pilar, 08006 Barcelona, Spain.

[5] Department of Clinical Microbiology, Hospital Clinic and School of Medicine, University of Barcelona, Barcelona, Spain.

[6] Barcelona Center for International Health Research (CRESIB), Hospital Clínic, University of Barcelona, Barcelona, Spain.

[7] Department of Infectious Diseases, Hospital Clínic and School of Medicine, University of Barcelona, Barcelona, Spain.

[8] Institut d'Investigacions Biomèdiques August Pi i Sunyer (IDIBAPS), University of Barcelona, Barcelona, Spain.

[9] ICFO-Institut de Ciencies Fotoniques, The Barcelona Institute of Science and Technology, 08860 Castelldefels (Barcelona), Spain.

[10] ICREA, Passeig Lluís Companys 23, 08010 Barcelona, Spain

* javier.garciadeabajo@icfo.es; Ramon.alvarez@urv.cat

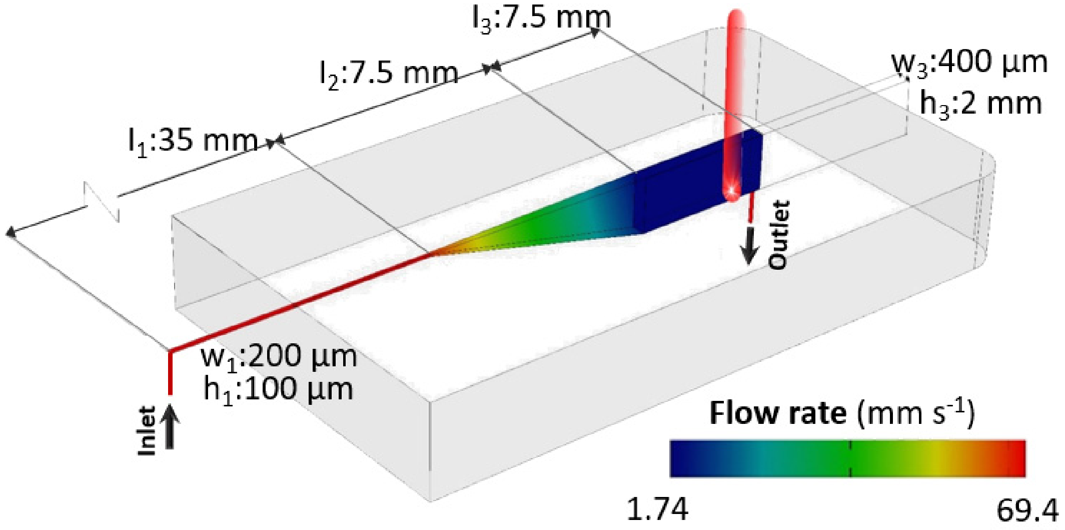

Figure S1. **Detailed schematics of the detection region in the millifluidic chipset of Figure 1.** The channel is divided in three sections: an initial narrow section used to increase pressure and reduce the flow to finely control the fluid discharge with the micropump; a second segment of increasing larger section; and, a third wide segment (0.4 mm width, 2 mm depth), with suitable dimensions to continuously screen the sample at flow rates of 1.74 mm s^{-1}, at a spectral capture rate of 1.83 mm s^{-1}.

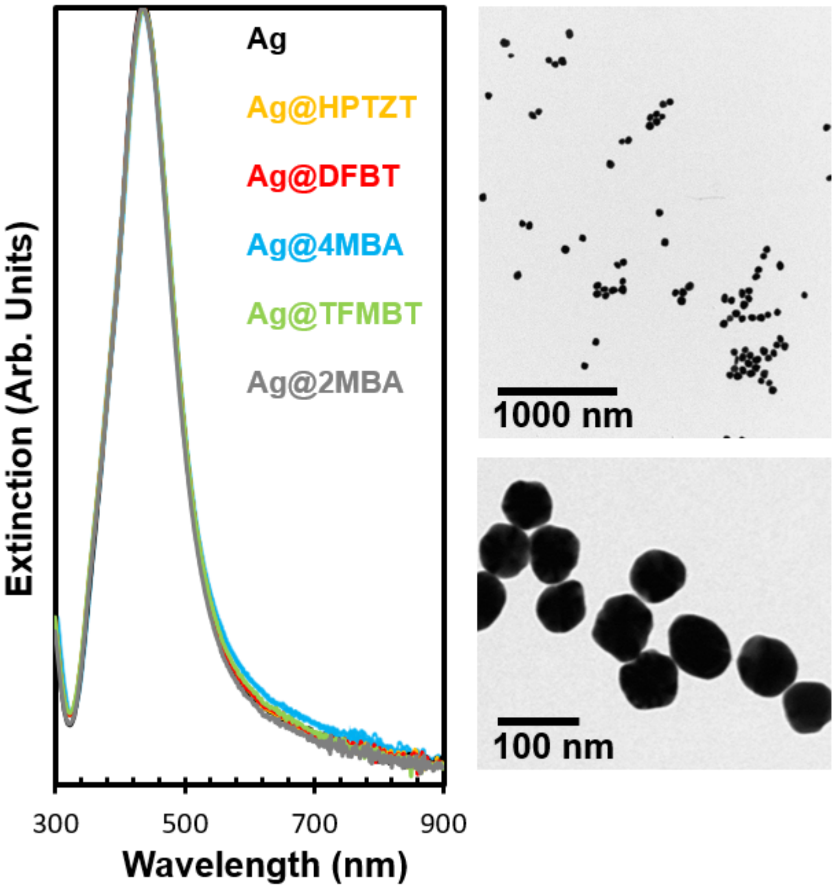

Figure S2. Optical and microscopy characterization of the NPs. Left: Optical extinction of functionalized Ag NPs in aqueous solution, showing a prominent plasmon consistent with the nominal diameter of 62 nm, and indicating that the coating molecules have a minor effect on the optical response. Right: Representative SEM images of as prepared encoded nanoparticles.

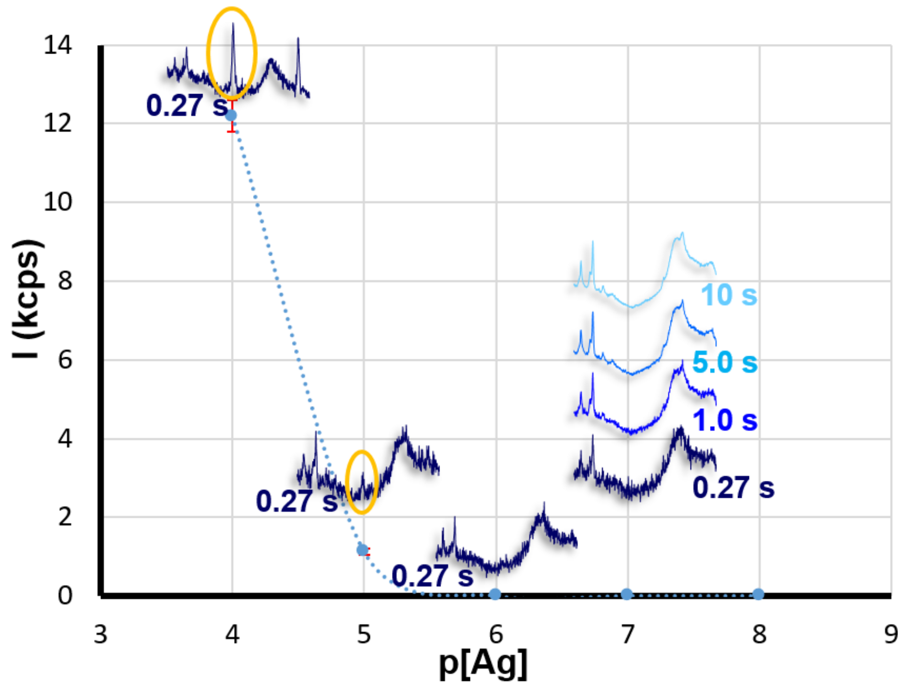

Figure S3. **Optimization of the concentration of encoded NPs for reduction of background noise.** We optimize the NP concentration to reduce the noise produced by the codes of dispersed NPs against the signal of decorated pathogens in the measurement area of the millifluidic chipset. We show SERS spectra of different dispersions of NPs arranged as a function of NP density (p is $-\log_{10}$ of the Ag molar concentration). A harmless, smooth, nearly constant background is produced by the chipset material. A SERS signal from the codes (circled features) can be clearly identified above 10^{-5} M in silver, but the spectrum disappears at 10^{-6} M. We operate at a silver concentration of 10^{-7} M (~10^{7} NPs per mL), for which no signal is achieved even after long acquisition times.

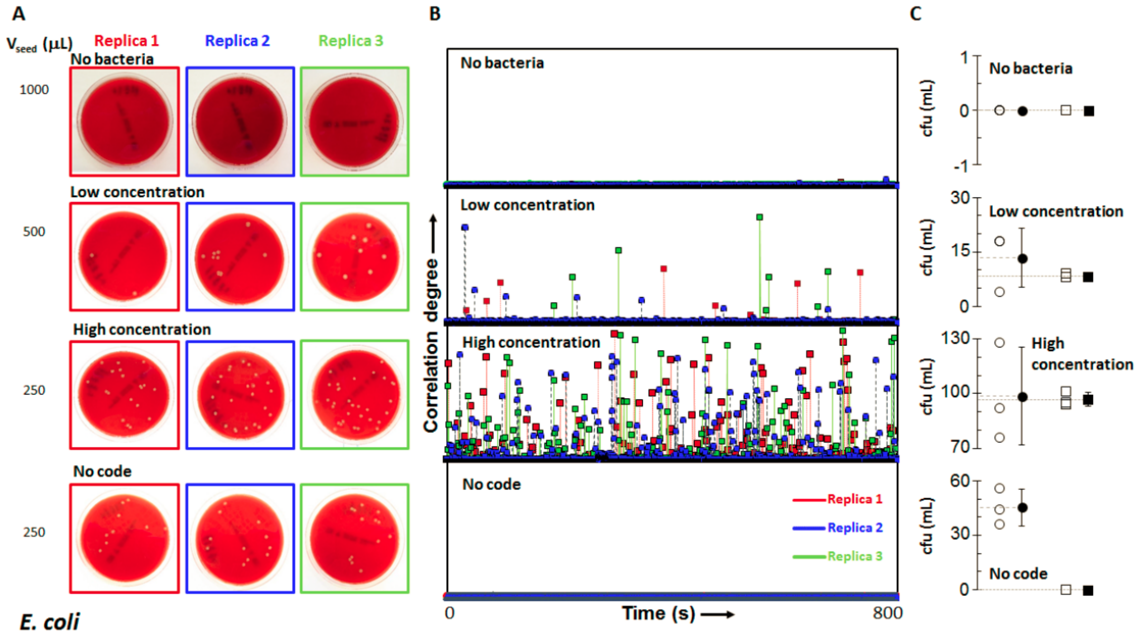

Figure S4. Results for serum sample spiked with *E. coli* at different concentrations. (A) Cellular cultures. **(B)** Detection and quantification results obtained in our MODS device, from top to bottom: With no bacteria present, at low pathogen concentration, high concentration, and high concentration but without the encoded nanoparticle that identifies these bacteria. **(C)** Statistical comparison of bacteria concentrations (CFUs per mL) as determined by MODS (open squares) versus traditional cultures (open circles). Averages over three runs of both MODS and culture experiments are shown by the corresponding solid symbols.

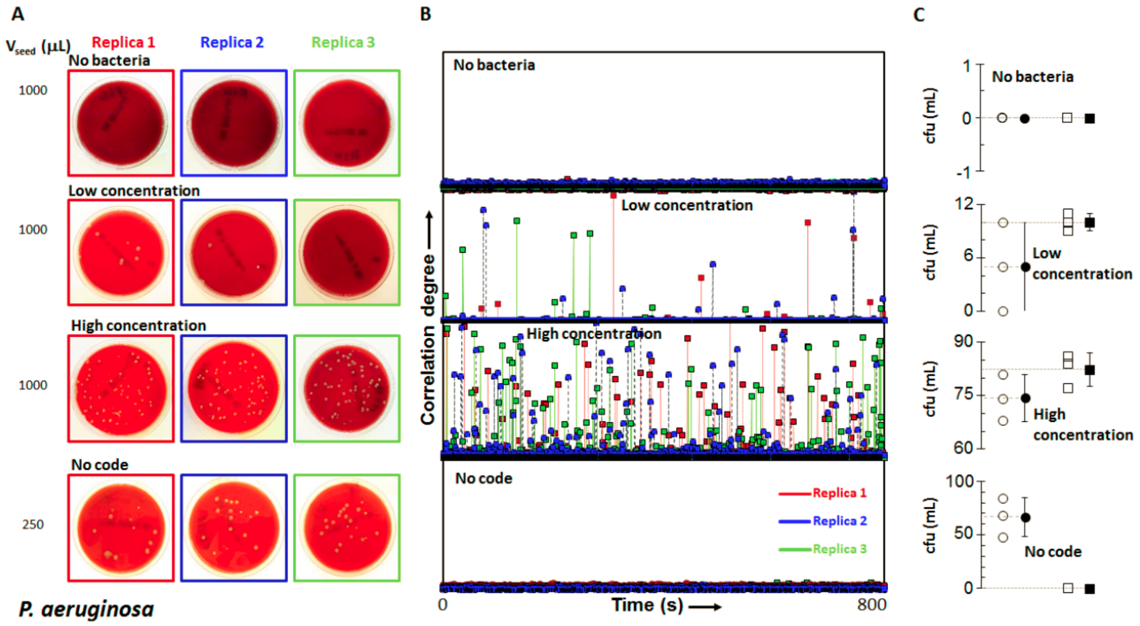

Figure S5. Same as Figure S4 for *P. aeruginosa* instead of *E. coli*.

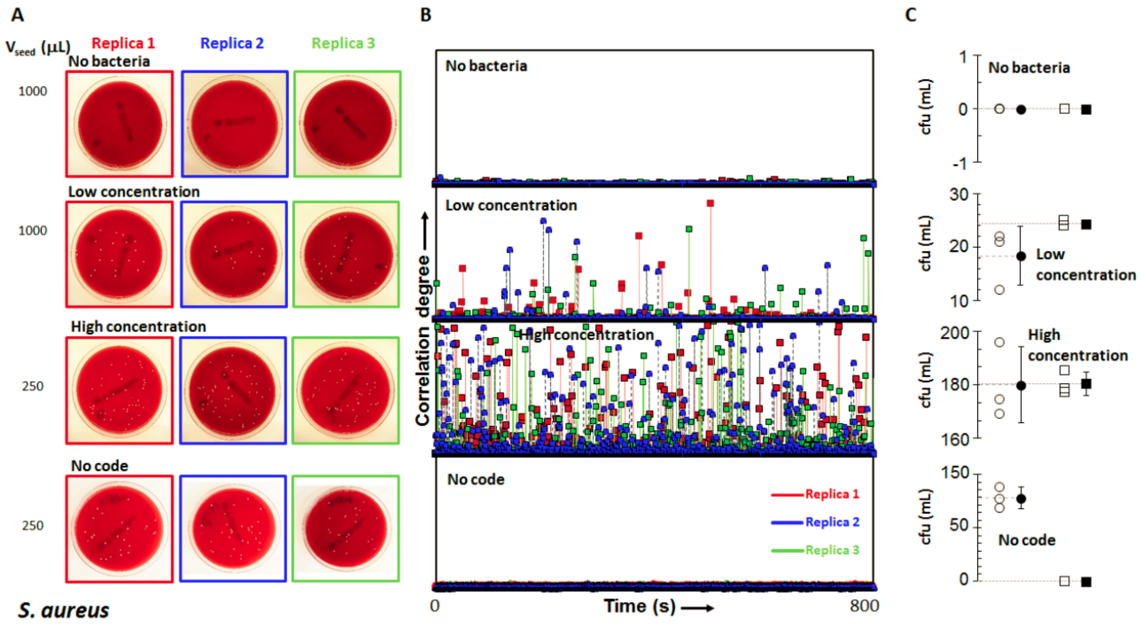

Figure S6. **Same as Figure S4 for *S. aureus* instead of *E. coli*.**

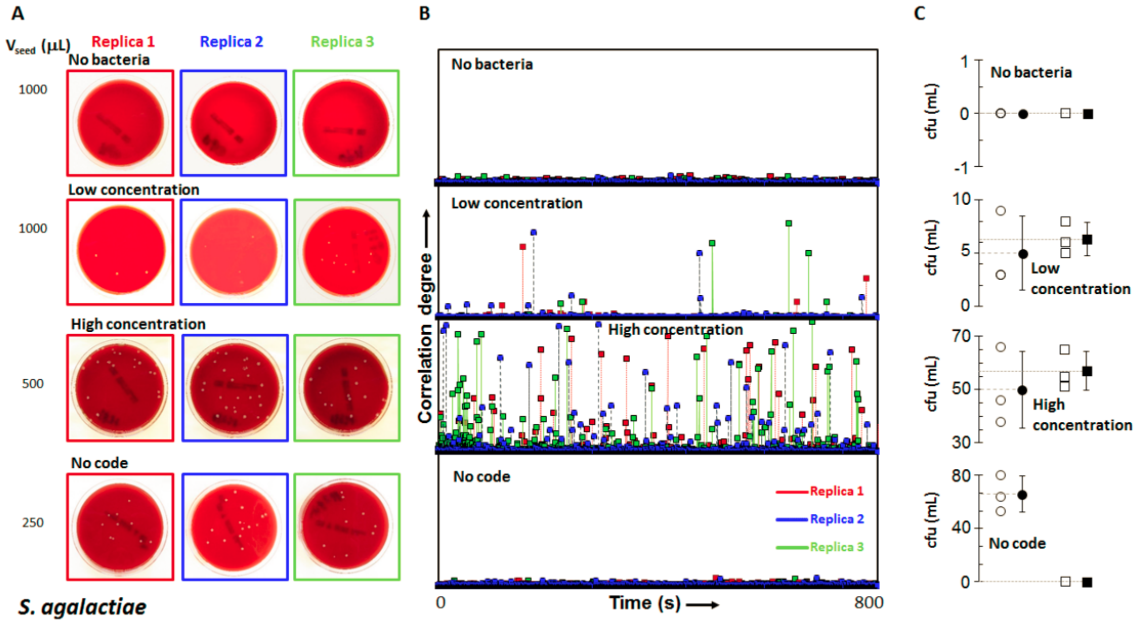

Figure S7. Same as Figure S4 for *S. agalactiae* instead of *E. coli*.

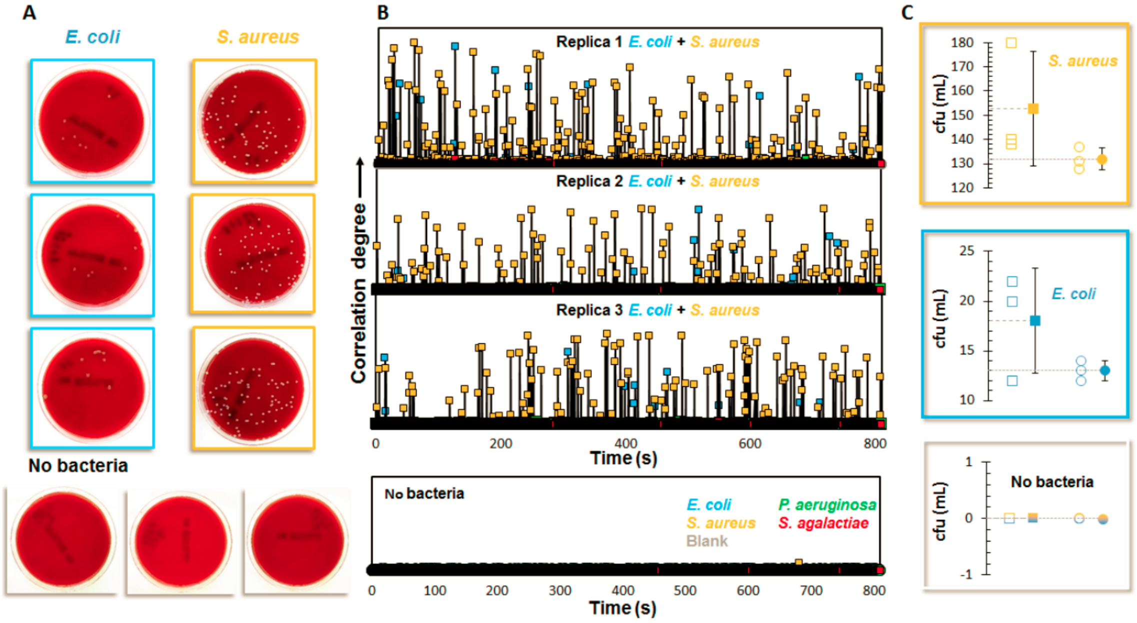

Figure S8. **Results for serum sample spiked with both *E. coli* and *S. aureus*. (A)** Cellular cultures for each bacteria (the volume seeded is 1 mL for E. coli and 0.5 mL for *S. aureus*). **(B)** Detection and quantification results obtained with MODS. **(C)** Statistical comparison of bacteria concentrations (CFUs per mL) as determined by MODS (open squares) versus traditional cultures (open circles). Averages over three runs of both MODS and culture experiments are shown by the corresponding solid symbols.

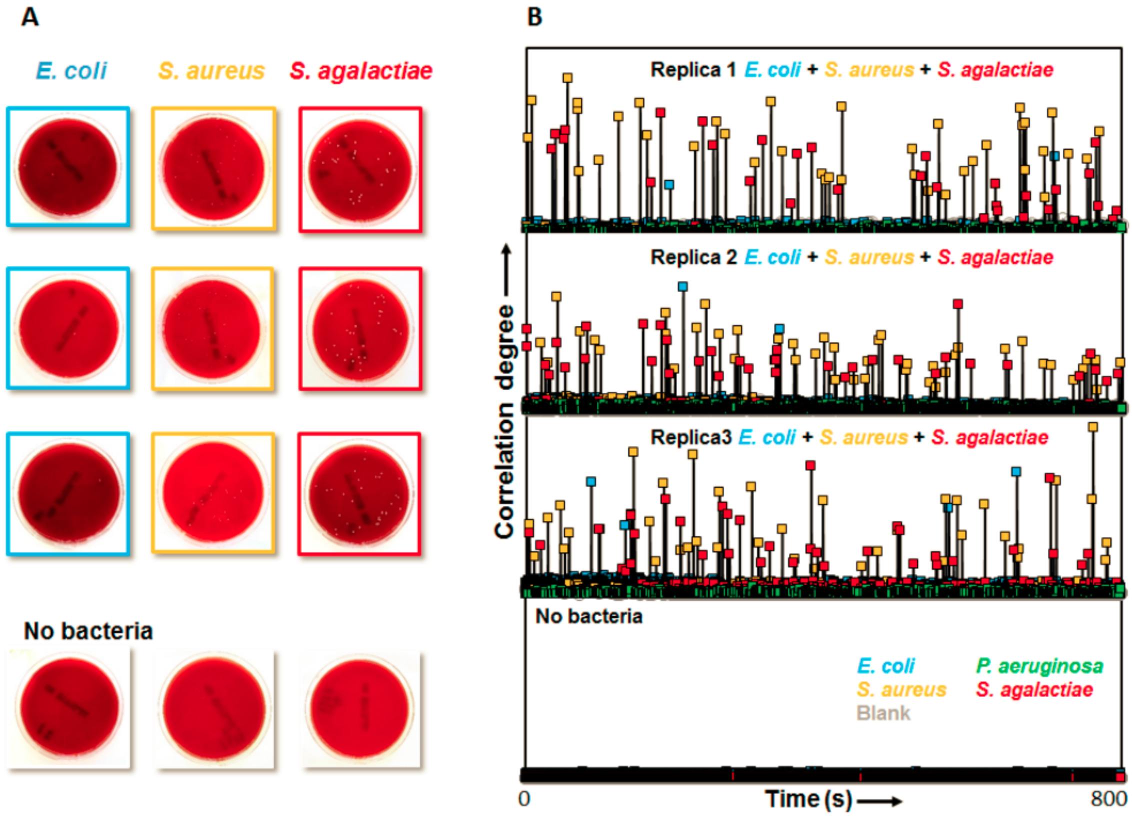

Figure S9. **Blood sample spiked with *E. coli, S. agalactiae*, and *S. aureus*. (A)** Cellular cultures for each bacteria (a volume 0.5 mL for *each*). **(B)** Detection and quantification results obtained in MODS. A statistical comparison of these results is presented in Figure 4C.

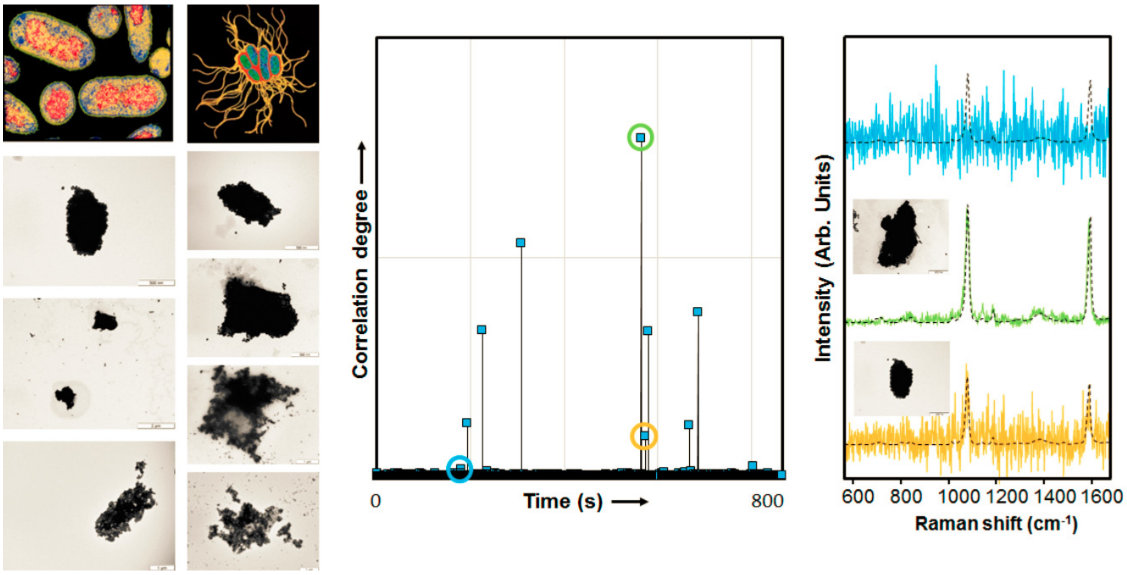

Figure S10. **Biodiversity of *E. coli*.** Left: TEM images of several single or clustered *E coli* CFU's coated with nanoparticles. Center: SERS detection results on the millifluidic MODS device for one of the samples of Figure S6 at low bacterial concentration. Right: Spectra corresponding to the events highlighted by circles in the previous plot, showing that the degree of correlation of the SERS spectra of the encoded particles strongly depends on the signal-to-noise ratio. Consequently, bigger clusters of bacteria give rise to larger signal and higher correlation.

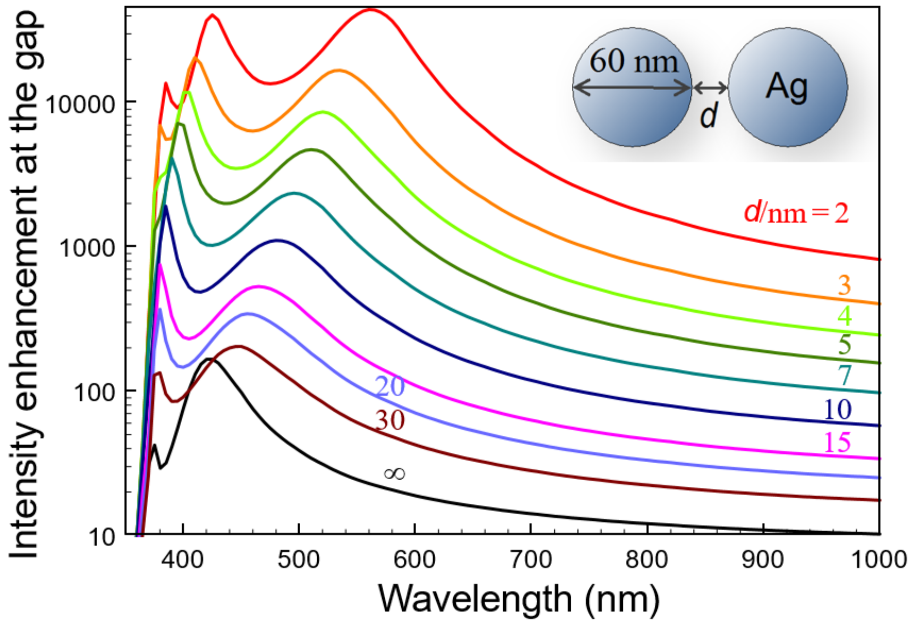

Figure S11. **Optical enhancement in Ag NP dimers.** We show the electric field intensity enhancement $|E/E^{ext}|^2$ generated by a dimer of silver nanoparticles (60 nm diameter) embedded in water (Ɛ=1.77). The intensity is calculated at a distance of 1 nm from the surface of one of the particles in the gap region. Different surface-to-surface separations *d* are considered (see labels). A significant intensity enhancement of several orders of magnitude is still observable at the off-resonance light wavelength of 785 nm used throughout this work.

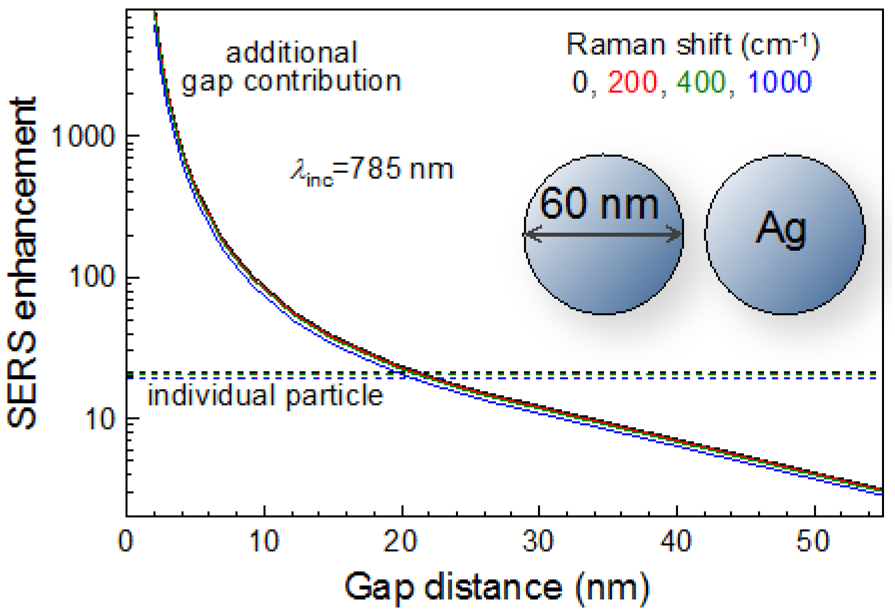

Figure S12. **SERS enhancement produced by Ag NP dimers in aqueous solution.** We show the increase in SERS enhancement factor due to inter-particle interaction (solid curves) compared with the values for individual particles (dashed lines) as a function of gap distance. This quantity is averaged over randomly distributed sampling molecules placed 1 nm outside the metal surface. An average over light polarizations and incidence directions is also carried out. The different Raman shifts under consideration (see labels) produce similar results because the incident light wavelength (785 nm) is not resonant with the particle plasmons (see Figure S11).

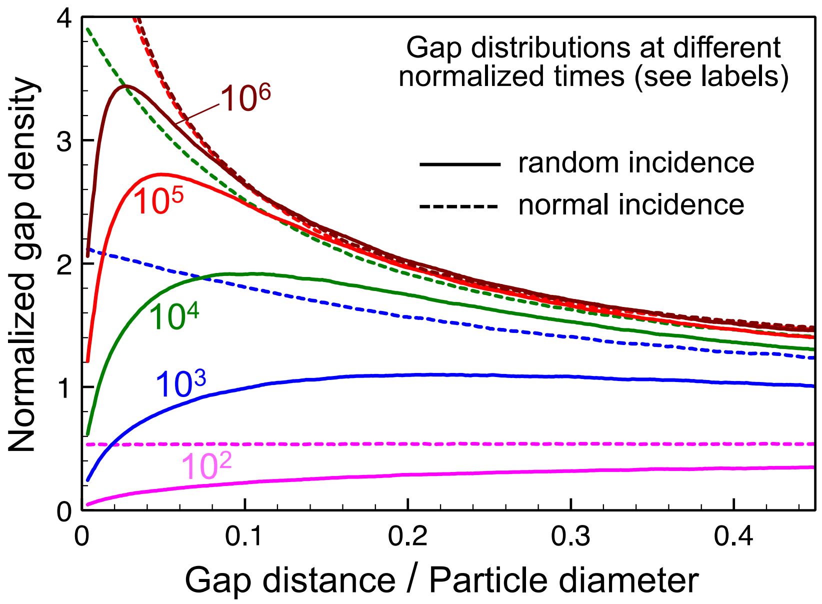

Figure S13. **Distribution of inter-particle gap distances.** We show the simulated distribution of gap distances between NPs attached on the pathogen membrane at different normalized times (see labels), both under random (solid curves) or normal (dashed curves) incidence directions relative to the membrane surface. The gap density distribution is normalized to the maximum NP density at regular close-packed coverage and it is given per unit of gap-to-diameter ratio. A time unit is defined here as the interval over which the number of particles colliding with the surface is equal to the number of attached particles under close-packed coverage. (See text above for more details.)

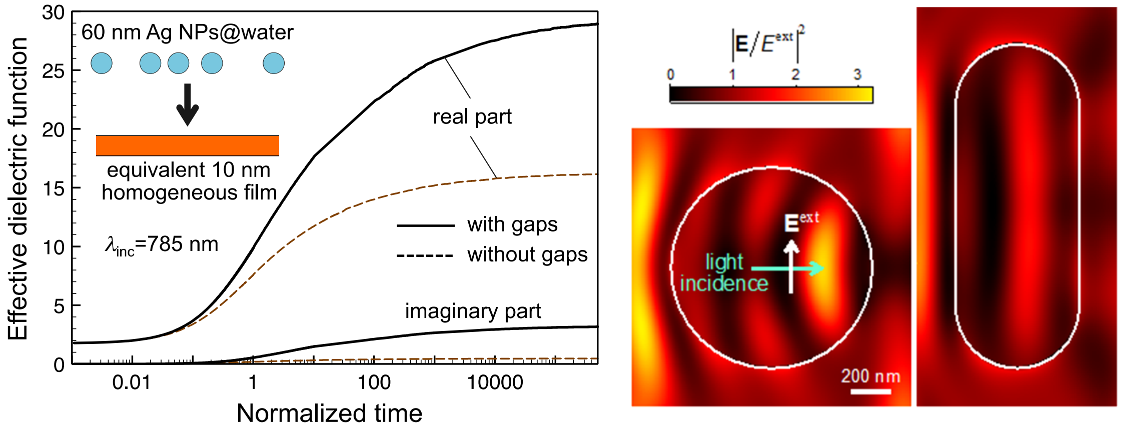

Figure S14. Near-field intensity in bacteria covered with nanoparticles. We assimilate the NP coating to a thin film with an effective dielectric function that is represented in the left plot. This dielectric function depends on the assumed film thickness (10 nm), but the calculated near field (right plot) is only mildly dependent on this parameter. The left plot shows that the dielectric function increases with the normalized time (see Figure S13 for time normalization), as a result of the increase in particle density. Similar to the SERS enhancement (see Figure 4B of the main paper), the gaps produce a large effect, creating additional polarization that further increases the dielectric function. The near-field intensity shown on the right density plots is calculated by replacing the NPs by an equivalent homogeneous film that is conformally covering sphere-like and rod-like microbes, respectively, with the media inside and outside the film both described as water (Ɛ=1.77). The light wavelength is 785 nm. Based on the results of the left plot, we model the coating as a 10 nm thick layer with permittivity equal to 28 + 3i. The intensity is observed to undergo relatively weak variations due to the coating, and therefore, the bulk of the SERS enhancement is produced by hotspots, as shown in Figure 4C.

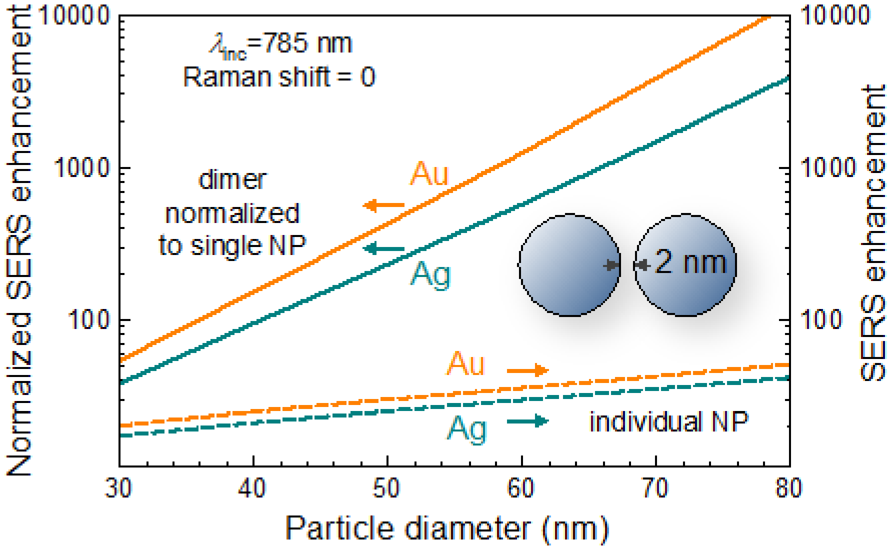

Figure S15. **SERS enhancement for Au and Ag dimers of different size.** We show the SERS enhancement averaged over molecule positions (1 nm outside the metal), light polarizations, and directions of incidence for gold and silver dimers as a function of particle size. The gap distance and the light wavelength are both fixed to 2 nm and 785 nm, respectively. The solid curves are the results for dimers normalized to the average over individual NPs (left vertical axis). The dashed curves show the absolute averaged enhancement for individual NPs (right vertical axis).
